# Neutrophil elastase in bronchiectasis

**DOI:** 10.1186/s12931-017-0691-x

**Published:** 2017-12-19

**Authors:** Andrea Gramegna, Francesco Amati, Leonardo Terranova, Giovanni Sotgiu, Paolo Tarsia, Daniela Miglietta, Maria Adelaide Calderazzo, Stefano Aliberti, Francesco Blasi

**Affiliations:** 1 Department of Pathophysiology and Transplantation, University of Milan, Internal Medicine Department, Respiratory Unit and Cystic Fibrosis Adult Center, Fondazione IRCCS Ca’ Granda Ospedale Maggiore Policlinico, Milan, Italy; 2 Department of Clinical Sciences and Community Health University of Milano, Paediatric Highly Intensive Care Unit, Fondazione IRCCS Ca’ Granda Ospedale Maggiore Policlinico, Milan, Italy; 3 Department of Biomedical Sciences, Clinical Epidemiology and Medical Statistics Unit, University of Sassari, Sassari, Italy; 4 Corporate Pre-Clinical R&D, Chiesi Farmaceutici SpA, Parma, Italy; 5 Global Clinical Development, Chiesi Farmaceutici SpA, Parma, Italy

**Keywords:** Bronchiectasis, Neutrophil elastase, Protease, Neutrophil elastase inhibitors, Desmosine

## Abstract

The role of neutrophil elastase (NE) is poorly understood in bronchiectasis because of the lack of preclinical data and so most of the assumptions made about NE inhibitor potential benefit is based on data from CF**.** In this context, NE seems to be a predictor of long-term clinical outcomes and a possible target of treatment. In order to better evaluate the role of NE in bronchiectasis, a systematic search of scientific evidence was performed.

Two investigators independently performed the search on PubMed and included studies published up to May 15, 2017 according to predefined criteria. A final pool of 31 studies was included in the systematic review, with a total of 2679 patients. For each paper data of interest were extracted and reported in table.

In this review sputum NE has proved useful as an inflammatory marker both in stable state bronchiectasis and during exacerbations and local or systemic antibiotic treatment. NE has also been associated with risk of exacerbation, time to next exacerbation and all-cause mortality. This study reviews also the role of NE as a specific target of treatment in bronchiectasis. Inhibition of NE is at a very early stage and future interventional studies should evaluate safety and efficacy for new molecules and formulations.

## Background

### Neutrophil elastase

Neutrophils play a key role in the pathogenesis of numerous diseases, ranging from chronic inflammatory disorders to infectious diseases and neoplasms [1]. Their role in the innate immune system mainly consists in extracellular phagocytosis, involving reactive oxygen species, cationic proteins, and enzymes. Several granules are sequentially formed in neutrophil cytoplasm, differing in their propensity for exocytosis, according to their biosynthesis timing. Granules formed at a later stage of differentiation shows a higher secretory potential than those formed in immature myeloid cells, such as the azurophilic granules containing myeloperoxidase, defensins, and proteinases. Neutrophils live shortly in the blood of healthy individuals (around 8 h), but following inflammatory conditions they become activated and their longevity may increase up to 5 days. The prolonged life of neutrophils leads to functional changes and contributes to the inflammatory-associated morbidity shown in bronchiectasis patients [2].

Once inflammatory trigger develops in peripheral tissues, neutrophils are rapidly recruited toward to the anatomical site of inflammation. Their migration out of the blood stream involves endothelial attachment and rolling along the endothelium, activation, adhesion, and, finally, trans-endothelial extravasation into the *interstitium*. The neutrophilic inflammatory response depends on the degranulation of pre-formed mediators from cytoplasmatic granules and on the ability to generate respiratory burst activity via reactive oxygen species system. Although they ontogenetically play key functions in antimicrobial defense, their activation can also damage host cells and tissues. Neutrophil degranulation is responsible for the release of several inflammatory mediators, such as neutrophil elastase, cathepsin G, and proteinase 3 [3].

Human neutrophil elastase (NE) is a proteolytic enzyme belonging to the chymotrypsin-like family of the serine-proteinases. NE is a 218 aminoacid long protein packaged in cytoplasmatic azurophilic granules in neutrophil granulocytes [4]. Though NE is mainly involved in the response against bacteria, it can cause several detrimental effects, including extracellular matrix destruction, mucus gland hyperplasia and increased mucus production, reduction of ciliary beating rate, and direct damage to the airway epithelium [5–7]. The acid *milieu* within granules protects the cell from proteolytic activity. Upon neutrophil activation, degranulation is triggered by the mild alkalosis resulting from the fusion between the granules and cytoplasmic phagosomes containing engulfed bacteria [8]. In addition to this intracellular pathway, NE is also liberated into the extracellular space, both free and membrane-bound. High concentrations of soluble NE participate in bacterial killing at the site of degranulation and in a small area around the cell. More distantly, activated enzymes are completely neutralized by proteinase inhibitors. On the other hand, membrane-bound NE is remarkably resistant to inhibition by the anti-proteinase system, thus preserving its proteolytic activity. Overexpression of this mechanism has the potential to cause tissue damage [9].

During neutrophil activation, NE-containing granules also translocate from the cytoplasm to the nucleus, where it is involved in the degradation of chromatin by splitting histones. Then, neutrophils disintegrate and release neutrophil extracellular traps (NETs). Although this remains a controversial or fully unexplored area, this process is hypothesized to facilitate the neutralization of pathogens, including bacteria, fungi, and protozoa. NETs are networks of extracellular fibers, composed of DNA bound to histone proteins and neutrophil proteinases. NE is the most common non-histone protein in NETs [10].

A complex interaction among proteases and anti-proteases is required for an effective respiratory immune system. Lower airways in patients with neutrophil elastase-mediated lung diseases are characterized by an excess of proteases leading to tissue destruction and lung function decline. A1AT is the most abundant anti-protease within the lung and the major inhibitor of NE. A1AT is encoded by the SERPINA1 gene located on the long arm of chromosome 14 and produced mainly by hepatocytes and, to a lesser extent, by alveolar macrophages. Imbalance between NE and A1AT may be due to either an A1AT deficiency, as documented in COPD and emphysema, or unopposed NE activity, as proved in CF and bronchiectasis [11]. In the latter context, NE shows the ability to combine with polyanions (such as DNA or glycosaminoglycans) and syndecan-1 becoming inaccessible and limiting its inactivation by A1AT [12].

Besides NE, the serine protease group also includes cathepsin G (Cat G) and proteinase 3 (PR3), which are also stored in high concentrations in the azurophil granules within the neutrophil cytoplasm [4]. Cat G is a 235-amino acid protein released during the neutrophil activation process. Intracellularly, Cat G is released within the phagosome to degrade engulfed pathogens; extracellularly, it contributes to the degradation of structural ECM components and other immune mediators [13]. PR3 is a 222-amino acid long serine protease encoded by the PRTN3 gene and expressed by activated neutrophils. Its biological function is similar to that of NE. In particular, PR3 is responsible for proteolytic degradation and activation of IL-8 in a truncated peptide with fostered chemoattractant effect towards neutrophils.

### NE determination assays

Different techniques have been used for NE determination. Both ready to use commercial kits and in-home assays are useful for in vitro quantitative determination of NE. The three most used techniques are ELISA [14], fluorimetric [15] and spectrophotometric [16] methods. In Table 1 some example of NE determination assays is provided showing different samples, types of measurement and detection ranges.

**Table 1:** Neutrophil elastase determination assays

Name	Samples	Type of measurement	Assay	Unit of measure	N° of test	Detection range	Limit of detection
Human Elastase ELISA (Hycult Biotech)	Plasma, Cell culture supernatant	Active NE	ELISA	ng/mL	2 × 96	0.4–25 ng/mL	0.4 ng/mL
ProteaseTag® Active NE Immunoassay (ProAxsis Ltd)	BAL, Sputum sol, Serum free media	Active NE	ELISA	ng/mL	96	15.625–1000 ng/mL	7.2 ng/mL
Human PMN Elastase ELISA Kit (Abcam)	Cell culture supernatant, Serum, Plasma	PMN Elastase/alpha1-PI complex	ELISA	ng/mL	96	0.16–10 ng/ml	1.98 pg/mL
Neutrophil elastase Human ELISA Kit, CE (eBioscience)	Cell culture supernatant, Serum, Plasma	PMN Elastase/alpha1-PI complex	ELISA	ng/mL	96	0.16–10.0 ng/mL	1.98 pg/mL
Neutrophil Elastase Activity Assay Kit (Abcam)	Plasma, Whole Blood, Purified protein	Active NE	Fluorometric	ng	100	1–25 ng	1 ng
Dynatech MR 5000 (Dynatech Corporation)	Sputum sol	Active NE	Spectrophotometry	mcgM	96	–	–
Neutrophil Elastase Activity Assay Kit (Cayman Chemical)	Blood	Active NE	Fluorometric	mU/mL	2 × 96	0–10 mU/mL	0.156 mU/mL

Definitions: *NE* neutrophil elastase, *alpha1-PI* alpha-1 proteinase inhibitor; *PMN* polymorphonuclear leukocytes

NE can be detected by two different methods: the assessment of NE ability to proteolytically cleave a synthetic substrate (qualitative measurement); and the quantification of human NE concentrations in different biological *media* (quantitative measurement). Both the detection of the active enzyme and the measurement of the amount of enzyme bound to its inhibitor, alpha-1 antitrypsin (A1AT), are techniques currently used to measure NE in biological samples.

When ELISA assays are employed, the wells in a microtiter plate are coated with a specific antibody able to bind NE. ProteaseTag® is a quantitative method to dose NE activity using NE-Tag and the subsequent antibody step provides additional signal amplification with increased sensitivity with good results in a recent study conducted by Chalmers [17].

Fluorimetric and spectrophotometric assays are based on the ability of NE to proteolytically cleave a synthetic substrate causing the release of a fluorophore or a change in optical density, respectively, which can be quantified by fluorescence or absorbance microplate readers. Standard curves are obtained for both methods and concentration of samples can be determined from curve interpretation.

Commercial kits may also be used to quantify NE in plasma, serum, cell-culture supernatant, bronchoalveolar lavage (BAL), sputum, or whole blood. Among other methodologies, elastin agarose diffusion plate for measurement of elastolytic activity has been also reported in previous literature [18].

Elastin degradation as a consequence of NE activity results in the presence of elastin-derived peptides, including desmosine and isodesmosine in serum, plasma and urine. Both separation based on high performance liquid chromatography/mass spectrometry and radioimmunoassay are validated techniques for the detection of iso- and desmosine in body fluids [19, 20]. Finally, high-performance capillary electrophoresis has been applied to the study of proteases [21].

## Neutrophil elastase in chronic respiratory diseases

### Cystic fibrosis

An outburst of neutrophilic inflammation characterizes CF patients and it is associated with high concentrations of neutrophil proteases, including NE. NE has several implications in inflammation in CF, although a comprehensive summary of NE effects is not in the purpose if this review NE has an important role in the pathogenesis of lung inflammation in CF even in the absence of any infections. High levels of both NE and IL-8 have been demonstrated in BAL of infants with CF [22]. A recent study by Sly showed a correlation between NE activity in BAL from CF children and the early development of bronchiectasis [23].

NE is a potent upregulator of several inflammatory chemokines, such as IL-8 and MMP-9, leading to a self-perpetuating cycle of neutrophilic inflammation with several detrimental effects [24]. Firstly, NE impairs ciliary beating and promotes expression of respiratory tract mucins (MUC5AC and MUC5B), resulting in muco-ciliary clearance failure [7, 25]. Secondly, NE-dependent structural damage against elastin and other pulmonary components leads to the irreversible airway dilation and early bronchiectasis development. Thirdly, NE can degradate lactoferrin, an important anti-microbial glycoprotein as well as several molecules involved in pathogens’ opsonization [26–29]. Furthermore, NE can cause impairment of lymphocytic function by degradation of T-cell surface receptors and interfere with the process of antigen presentation by dendritic cells [30, 31]. The sum of all these mechanisms leads to the paradox of an overstimulated immune system unable to effectively kill colonizing pathogens.

Finally, NE may directly affect the ion transport in CF cells acting as a potent activator of silent ENaC channels causing increased Na absorption and further airway surface dehydration and muco-ciliary dysfunction [32]. In a recent study, Le Gars and coworkers illustrated that high NE levels lead to CFTR loss of function via activation of intracellular calpains that are directly responsible for the proteolytic degradation of CFTR [33].

Treatments targeted against NE have been evaluated in CF patients. AZD9668, a reversible oral inhibitor of NE, when administered 60 mg twice daily orally for 4 weeks, showed no effect on sputum neutrophil counts, NE activity, lung function or clinical outcomes, but a consistent pattern of reduction in sputum inflammatory biomarkers and a significant decrease of free and total urine desmosine were reported in a double-blind randomized controlled trial (RCT) enrolling ~60 CF patients [34]. A phase-II clinical trial assessed the efficacy of inhaled A1AT, with significant reduction of NE activity and inflammatory biomarker expression without any effects on lung function [35]. The limited efficacy observed with both AZD9668 and A1AT in CF patients could be mainly ascribed to the fact that the studies were most likely too short and not powered to investigate disease-modifying effects.

### Chronic obstructive pulmonary disease

In patients with COPD neutrophils in sputum correlate with severity of disease in terms of FEV_1_ decline and peripheral airway dysfunction identified by high-resolution computed tomography [36]. Several studies highlighted that cigarette smoke and lung pollutants activate macrophages and epithelial cells triggering the release of neutrophil chemotactic factors [37, 38]. In this context NE is a major determinant of tissue damage. A landmark study on COPD-related lung tissue by Damiano showed a positive correlation between the distribution of NE within alveolar spaces and the presence of emphysema [39]. Instillation of human NE has been shown to induce emphysema in the animal model, while Shapiro and coworkers demonstrated that mice deficient for NE expression were protected from the development of emphysematous changes [40, 41]. More recently, Paone have found that elevated levels of NE in COPD in both sputum and BAL highly correlated with severity of disease and lung function decline [42].

Treatment strategies targeting NE have been proposed also for patients with COPD. AZD9668 was shown to slow lung function decline and dampen the inflammatory burden [43]. However, two RCTs which recruited symptomatic COPD patients exposed to AZD9668 for 12 weeks failed to show any clinical efficacy nor beneficial effects on inflammatory biomarker expression [44, 45]. This lack of efficacy may be due to inappropriate study design in term of patients selection, relevant dose, duration of treatment and endpoints assessments. It has been recently demonstrated that in a subpopulation with alpha1-antitrypsin (A1AT) deficiency, intravenous administration of A1AT slowed the loss of lung parenchyma [46].

### Asthma

Chronic inflammation in asthma is highly heterogeneous. Although in the past asthma was thought as a mere Th2-mediated inflammatory response to inhaled antigens, neutrophils and their products have recently been considered to play a role during both stable conditions and exacerbations [47, 48]. Simpson and colleagues showed that neutrophilic asthma is characterized by higher concentrations of active NE, generating the hypothesis that anti-proteases may be beneficial in the treatment of asthmatic patients [49]. At the current state, no studies on NE inhibitors in patients with asthma are currently available.

## Neutrophil Elastase in Bronchiectasis

Bronchiectasis is defined as an abnormal and permanent dilation of the bronchi associated with daily cough, sputum production, and recurrent respiratory infections [50]. The prevalence of bronchiectasis has risen during the last decade both in hospital and community settings owing to population aging, as well as widespread use of chest CT scan and more awareness of the disease [50, 51]. The pathophysiology of bronchiectasis is not completely understood, particularly in view of the heterogeneity of the disease and the absence of an animal model [52]. A fundamental role is played by both impaired mucus clearance and chronic bacterial infections leading to a serious neutrophilic activation by the release of chemotactic mediators. A recent study by Dente and coworkers demonstrated that sputum neutrophils in bronchiectasis patients correlated with worse pulmonary function, bacterial colonization, and severe disease [53]. In this context, NE is a key determinant of tissue damage and a potential marker of both disease severity and activity, a predictor of long-term clinical outcomes, and a treatment target. In order to better evaluate the role of NE in bronchiectasis, a systematic search of scientific evidence on NE in bronchiectasis was performed.

## Methods

### Search methodology

Two investigators (AG and FA) independently performed the search on PubMed and assessed the studies according to predefined criteria. Reference lists of the selected manuscripts were also manually assessed. English language restriction was applied. This systematic revision was conducted according to PRISMA statement [54].

#### Study selection

We included studies published up to May 15, 2017. Key terms included: ‘bronchiectasis AND neutrophilic elastase OR neutrophil elastase’; ‘bronchiectasis AND protease’; ‘bronchiectasis AND neutrophil elastase inhibitors’; ‘bronchiectasis AND desmosine’. After literature search, titles and abstracts were reviewed by two independent investigators (AG and FA) and in case of disagreement a final decision was taken by the principal investigator (SA). Articles were excluded if: (1) written in languages other than English; (2) they were case reports, case series, or qualitative reviews; (3) NE was not measured; (4) missing bronchiectasis patients; (5) inclusion of CF patients; (6) inclusion of pediatric patients; (7) full-text was unavailable. Full-text was finally obtained for selected papers.

#### Data extraction and analysis

After the full-text analysis, data of interest from each included paper were extracted. Data of interest included: name of the first author, year of publication, study design, sample size, assay manufacturer, biological *matrix*, setting, clinical state of patients, quantitative findings and endpoints. Corresponding authors were contacted if data were not present or unclear in the full-text. Given the high degree of heterogeneity across papers taken into account, a meta-analysis was not performed.

## Results

**Fig. 1:**
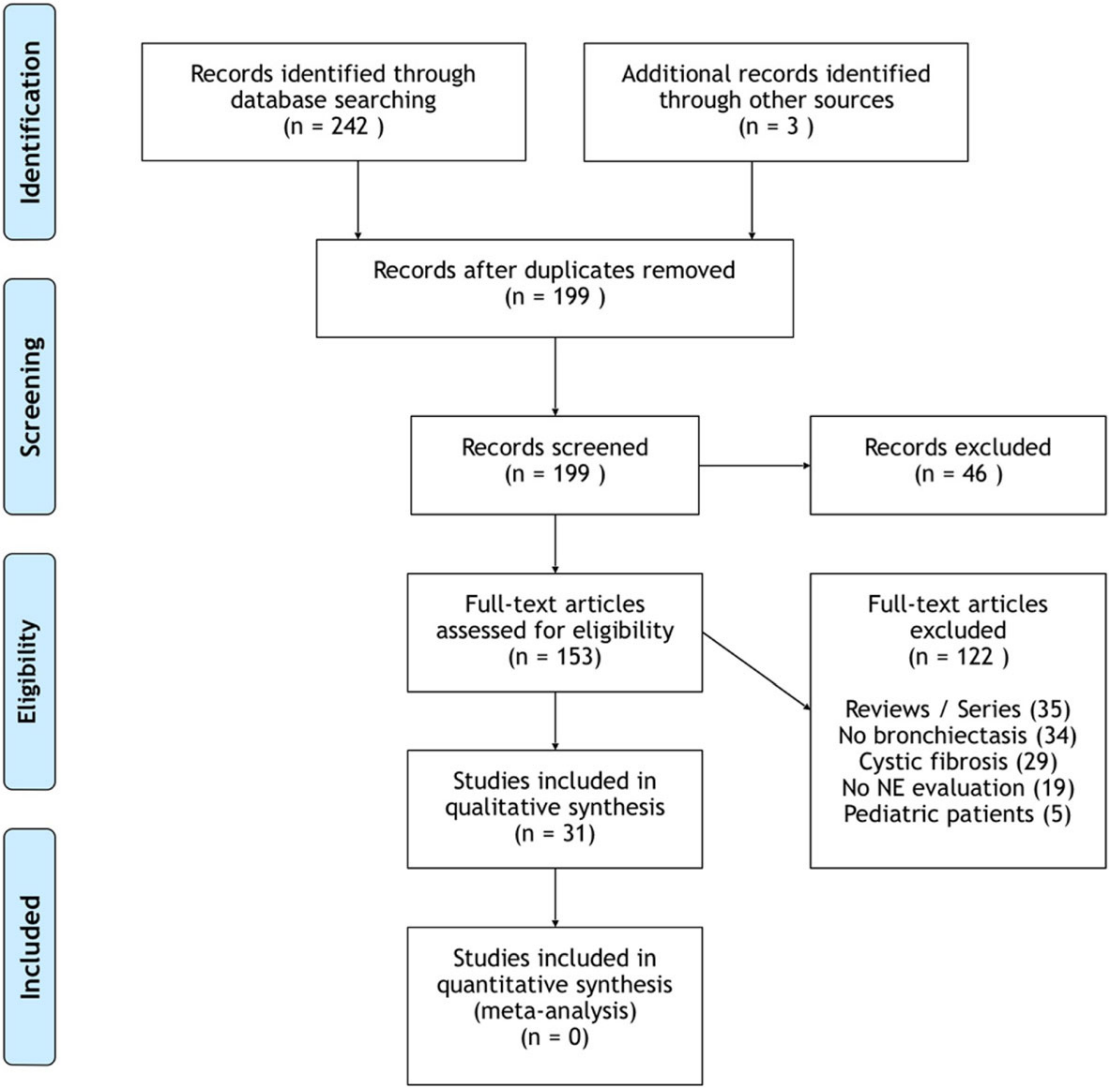
Flow-chart of the systematic review

The process and results of the search are shown in Fig. 1. The majority of the selected studies were rejected because either they were qualitative reviews or case series (*n* = 35) or not dealing with bronchiectasis patients (*n* = 34), or evaluating CF patients (*n* = 29) /children (*n* = 5), or their full-text was not available in English (*n* = 16). A final pool of 31 studies was included in the systematic review, with a total of 2679 patients (Table 2). Selected papers were published from 1984 to 2016, with an elevated frequency in the period 1998–2002 (10/31, 32.3%). The majority had a cross-sectional design (19/31, 61.3%). Only six (19.4%) studies included patients during an acute exacerbation. Measurement of NE was highly heterogeneous, including both commercial kits and in-home assays. Sputum was the most represented biological sample for NE detection. Blood samples were occasionally collected for NE or desmosine measurement [17, 21, 55]. Only one (3.2%) study detected urine desmosine. Most of the selected studies were aimed to assess NE as an inflammatory marker (19/31, 61.3%), while 7 (22.6%) studies investigated NE as a marker of response to treatment and 1 (3.2%) study evaluated NE as a target of therapy.

**Table 2:** Results of the systematic review

Author and year	Study design	Setting	Clinical stability	Sample size	Assay manufacturer (methods)	Quantitative findings	Biological *matrix*	Major results
Stockley, 1984 [18]	Cross-sectional	Monocentric, outpatients	Stable state Exacerbation	34 Bx patients	Elastin-agarose diffusion plate (in-home assay)	NE activity (μg x mL): grade1 19.5; grade2 48.2; grade3 62.9	Sputum	Correlation with sputum macroscopic appearance
Smallman, 1984 [6]	Cross-sectional/Prospective	Monocentric, outpatients	Stable state	8 Bx patients	Elastin-agarose diffusion plate (in-home assay)	NE activity (μg x mL): not quantified but evaluated as present/not present (lower limit of detection 0.8)	Sputum	Inverse correlation with ciliary beat frequency NE reduction after short-term antibiotic therapy
Fujita, 1992 [60]	Cross-sectional	Monocentric, outpatients	Stable state	9 Bx patients in a cohort of 64 chronic respiratory diseases +15 healthy subjects	Elisa for NE- A1AT complex (in-home assay)	NE-A1AT complex levels (μg x L): 558 ± 198; healthy subjects 122 ± 4	Blood	NE- A1AT complex higher in Bx patients than in healthy subjects
Lloberes, 1992 [63]	Cross- sectional	Monocentric, outpatients	Stable state	26 Bx patients	Elastin-agarose diffusion plate (in-home assay)	NE activity (μg × 100 μL): purulent 7.75 (0–21.5); mucopurulent 1.3 (0–19.5); mucoid 0	Sputum	Correlation with sputum macroscopic appearance
Ip, 1993 [71]	Prospective	Monocentric, outpatients	Exacerbation	12 Bx patients	Spectrophotometry, SLAPN substrate	NE activity (mU × 100 μL): baseline 50.5 ± 17.1; exacerbation before atb 51.8 ± 25.4; 1-week atb −24.3 ± 20.7; 2-week atb −35.1 ± 17.8; 2-week after atb −12.1 ± 35.6; 6-week after atb −32.8 ± 26.8	Sputum	NE reduction after short-term antibiotic therapy
Llewellyn-Jones, 1995 [15]	Prospective	Monocentric, outpatients	Stable state	9 Bx patients +8 healthy subjects	Fluorescence (in-home assay)	NE activity (μg x mL^−1^): day -7th 4.0 ± 0.46; baseline 4.15 ± 0.69; day 14th 4.25 ± 0.63; day 28th 4.39 ± 0.65; day 63th 4.03 ± 0.67	Blood	No NE reduction after indomethacin
Sepper, 1995 [74]	Cross-sectional	Monocentric, outpatients	Stable state	24 Bx patients +15 healthy subjects	Spectrophotometry, SAAVNA substrate	NE activity (mU x g): mild group 0.21 ± 0.09; moderate group 1.87 ± 1.12; severe group 2.64 ± 1.63; healthy subjects 0.09 ± 0.03	BAL	Correlation with symptoms, exacerbation rate and radiological severity
Nakamura, 1997 [56]	Cross-sectional	Monocentric, outpatients	Stable state	3 Bx patients in a cohort of 8 chronic respiratory diseases +15 healthy subjects	Elisa (in-home assay)	Quantitative findings only reported as figures	BAL	NE higher in Bx than in healthy subjects
Gaga, 1998 [57]	Cross-sectional	Monocentric, outpatients	Stable state	12 Bx patients +11 healthy subjects	Immunocitochemistry (in-home assay)	Neutrophils (cells x mm^3^): Bx patients 114; healthy subjects 41	Bronchial biopsies	NE higher in Bx than in healthy subjects
Nakamura, 1999 [78]	Prospective	Monocentric, outpatients	Stable state	3 Bx patients in a cohort of 10 chronic respiratory diseases	Elisa (in-home assay)	NE levels (μg x mL^−1^): baseline 125.5 ± 47.5; 16.8 ± 7.1 after atb	BAL	Ne reduction after 3-month roxithromycin
Ichikawa, 1999 [64]	Cross-sectional	Monocentric, outpatients	Stable state	18 Bx patients in a cohort of 59 chronic respiratory diseases +28 healthy subjects	Spectrofluorometry AMC substrate	NE activity (U): purulent 1239.2 ± 1017.8; mucoid 45.7 ± 115.1	Sputum	Inverse correlation with IgBF levels and sputum purulence
Hill, 2000 [16]	Cross- sectional	Monocentric, outpatients	Stable state	43 Bx patients in a cohort of 160 chronic respiratory diseases	Spectrophotometry	NE levels (nM): mixed flora 0 (0–20); high bacterial load 21 (4–40)	Sputum	Correlation with sputum bacterial load
Schaaf, 2000 [68]	Cross-sectional	Monocentric, outpatients	Stable state	11 Bx patients in a cohort of 66 chronic respiratory diseases +12 healthy subjects	Immunoluminometry Colorimetry	NE-A1AT complex levels (ng x mL): Bx patients 44 (15–152); healthy subjects 3 (0–15)	BAL	NE higher in Bx than in healthy subjects Correlation with *P. aeruginosa* chronic respiratory infection
Tsang, 2000 [75]	Cross-sectional	Monocentric, outpatients	Stable state	30 Bx patients	Spectrophotometry, SAAVNA substrate	NE levels (U x mL): 6.60 ± 3.13	Sputum	Correlation with 24 h sputum volume, sputum inflammatory markers (leukocytes, IL-B and TNFa), radiological severity and spirometry (FEV_1_, FVC)
Zheng, 2000 [69]	Cross-sectional	Monocentric, outpatients	Stable state	35 Bx patients +18 healthy subjects	Spectrophotometry, SAAVNA substrate	NE levels (Unit x mL^−1^): P. aeruginosa 222.6 (18.3–267.5); 8.7 (0.6–186.7)	Sputum	Correlation with *P. aeruginosa* chronic respiratory infection
Viglio, 2000 [21]	Cross-sectional	Monocentric, outpatients	Stable state	13 Bx patients in a cohort of 54 chronic respiratory diseases +24 healthy subjects	HPCE	DES (μg x g creatinine^−1^): Bx patients 23.39 ± 2.05; CF 23.39 ± 2.02; A1AT deficiency 22.3 ± 7.74; exacerbated COPD 17.15 ± 3.42; stable COPD 14.17 ± 2.33; smokers 11.97 ± 2.75; healthy subjects 9.31 ± 2.75	Urine	Desmosine higher in stable Bx than in other chronic respiratory disease and healthy subjects
Angrill, 2001 [62]	Cross-sectional	Monocentric, outpatients	Stable state	49 Bx patients +9 healthy subjects	Immunoassay (?)	NE levels (μg x L): colonized patients 231 (15–2930); not colonized patients 45 (8–2280); healthy subjects 34 (9–44)	BAL	NE higher in Bx than in healthy subjects Correlation with neutrophils, IL8 and TNFa in BALF Correlation with bacterial chronic respiratory infection.
Stockley, 2001 [65]	Cross-sectional	Monocentric, outpatients	Stable state	14 Bx patients +9 smokers	Spectrophotometry, MSAPN substrate Elisa (in-home assay)	NE levels (μM): sputum colour value-3 0.006 ± 1.0; sputum colour value-7 4.14 ± 1.0	Sputum	Correlation with sputum macroscopic appearance Correlation with MPO and 24 h sputum volume Inverse correlation with A1AT
Zheng, 2001 [59]	Cross-sectional	Monocentric, outpatients	Stable state	14 Bx patients +15 healthy subjects	Anti-NE antibodies	Median neutrophils x mm^−2^: Bx patients 608 (101–1013); healthy subj. 127 (24–630)	Endobronchial biopsies	NE higher in bronchiectatic lamina propria than in healthy subjects
Vandivier, 2002 [58]	Prospective	Monocentric, outpatients	Exacerbation	6 Bx patients in a cohort of 18 chronic respiratory diseases	Spectrophotometry	NE (U x mL): Bx patients 10; CF 3	Sputum	NE higher in Bx NE as cause of delay in apoptotic cell clearance
Chan, 2003 [12]	Cross-sectional	Monocentric, outpatients	Stable state	10 Bx patients	Spectrophotometry	NE activity range (mM): 0.9–1.2	Sputum	NE not only as free but complexed with polyanionic partners (syndecan-1) and A1AT
Watt, 2004 [72]	Prospective	Monocentric, outpatients	Exacerbation	15 Bx patients +10 healthy subjects	Kinetic assays (?)	NE levels (ng x mL): after atb −73,451 (135,495–12,303)	Sputum	NE reduction after short-term antibiotic therapy
Chan, 2009 [61]	Cross-sectional	Monocentric, outpatients	Stable state	12 Bx patients	Spectrophotometry, MSAPN substrate ELISA (in-home assay)	NE activity (mM): 1.3 (1–2)	Sputum	NE complexed with syndecan-1 Description of protease/anti-protease balance
Murray, 2010 [77]	RCT	Monocentric, outpatients	Stable state	65 Bx patients	Elastin-agarose diffusion plate (in-home assay)	NE activity gentamicin group vs placebo: (U x mg): baseline 3.6 (0–17.6) vs 4.1 (0–19); 3-month 0 (0–0) vs 0 (0–20.4); 6-month 0 (0–2.9) vs 0 (0–29.1); 9-month 0 (0–7.6) vs 0.9 (0–19.4); 12- month 0 (0–1.8) vs 1.8 (0.17–16); 15-month 7.1 (0–56) vs 2.8 (0.9–18.2)	Sputum	NE reduction after 3-month inhaled gentamicin
Chalmers, 2012 [67]	Prospective	Monocentric, outpatients	Stable state Exacerbation	385 Bx patients	Elastin-agarose diffusion plate (in-home assay)	NE activity (μg x mL): P. aeruginosa infected 4.9 (1.2–68); P. aeruginosa not infected 1.2 (0.4–147.2); other quantitative findings only reported as figures	Sputum	Correlation with bacterial load and radiological severity
Mandall, 2013 [73]	Prospective	Monocentric, outpatients	Stable state	163 Bx patients	Spectrophotometry	NE activity (U x mL): GERD group 5; non-GERD group 3	Sputum	Correlation with GERD
Stockley, 2013 [55]	RCT	Multicentric, outpatients	Stable state	38 Bx patients	Custom-made immunoassay (in-home assay) for both NE and desmosine	NE activity AZD9668 group vs placebo (μM x L): baseline 24.54 vs 7.28; end of treatment 15.92 vs 7.49	Sputum Urine	NE reduction after 28-day of oral AZD9668 No difference in desmosine between AZD9668 and placebo
Goeminne, 2014 [66]	Cross-sectional	Monocentric, outpatients	Stable state	49 Bx patients +12 healthy subjects	Enzymatic assay	NE activity (μg x mL): Bx patients 15 (3–23); healthy subject 0.8 (0.4–1.2)	Sputum	Correlation with TNFA, CXCL8 and MMP-9 Inverse correlation with FVC
Liu, 2014 [14]	RCT	Monocentric, outpatients	Stable state	43 Bx patients	ELISA (in-home assay)	Quantitative findings only reported as figures	Sputum	NE reduction after 6-month roxythromicin
Aliberti, 2016 [70]	Prospective	Multicentric, outpatients	Stable state	1145 Bx patients	different assays according to different centers	Quantitative findings only reported as figures	Sputum	Correlation with ‘*P. aeruginosa‘*cluster and ‘Other chronic infection’ cluster
Chalmers, 2016 [17]	Prospective		Stable state Exacerbation	381 Bx patients	Immunoassay (ProAxsis LTD) Kinetic assay (Sigma Aldrich) LC-MS for desmosine	NE levels (μg x mL): baseline 0.39 (0–23.5); onset of exacerbation 57.0 (3.3–145); after 14-day atb 0 (0–125.8); 1-month later 1.3 (0–29.9)	Sputum Serum	Correlation with BSI, MRC, FEV_1_, bacterial load, radiological severity Correlation with higher exacerbation rate, FEV1 decline, all-cause mortality during 3-year follow up NE increase during exacerbations and NE reduction after short-term antibiotic therapy Serum desmosine is associated with age and disease severity

Definitions: *atb* antibiotics, *A1AT* alpha-1 anti-trypsin, *BAL* broncho-alveolar lavage, *Bx* bronchiectasis, *DES* desmosine, *NE* neutrophil elastase, *SLAPN* succinyl-trialanine-nitroanilide, *SAAVNA* succinyl-Ala-Ala-Val-nitroanilide, *MSAPN* methoxysuccinyl-Ala-Ala-Pro-Val-paranitroanilide, *HPCE* high-performance capillary electrophoresis, *LC-MS* liquid chromatography-mass spectrometry

## Discussion

Clinical relevance of NE role in bronchiectasis has been addressed according to a multidimensional approach: its use as inflammatory biomarkers; potential indicator of severity of disease; predictor of clinical outcomes or response to treatment; target of treatment.

### NE as an inflammatory marker

Patients with clinically stable bronchiectasis exhibit a permanent neutrophilic activation in the airways and display higher sputum levels of NE and others inflammatory mediators than healthy subjects [56–58]. Because of the presence on NE-positive neutrophils in the *lamina propria*, the inflammation is assumed to be driven by NE [59].

Fujita and Chan demonstrated in different studies that serum levels of NE-A1AT complex in patients with bronchiectasis were significantly higher in comparison to those of healthy subjects suggesting that lung injury is mainly due to NE overexpression than anti-proteinase system deficiency [12, 60, 61].

Angrill proposed that airway inflammation is permanent and may occur even in the absence of bacterial colonization, as demonstrated by the increase of NE and other inflammatory mediators in the BAL of 49 patients in comparison with healthy subjects [62]. Previous studies reported that purulent sputum was associated with NE concentration, and can be considered a marker for proteolytic and inflammatory activity [18, 63–65]. These findings were consistent with recent data reported by Goeminne who demonstrated a strong correlation between sputum purulence and proteolytic enzymes, both of which seem to predict the degree of inflammation and disease severity in bronchiectasis [66].

Moreover, NE has been shown to progressively increase with increasing bacterial load in sputum. Chalmers also reported on a strong association between sputum bacterial load and a cluster of inflammatory mediators in a cohort of 434 bronchiectasis patients [16, 67]. Finally, high levels of NE have been documented in special phenotypes of bronchiectasis patients, such as those with chronic infection with both *P. aeruginosa* and other bacteria [68–70].

As a marker of airway inflammation, NE also increases during exacerbations and decreases after a short course of oral antibiotic treatment [6, 67, 71, 72]. These data suggest that NE may act as marker of airway inflammation both in stable patients and during exacerbations.

A correlation between gastroesophageal reflux disease (GERD) and higher levels of NE is also reported [73].

### NE as a predictor of clinical outcomes or response to treatment

The identification of a biomarker to assess disease severity and predict progression and outcomes is still an unmet need in bronchiectasis [51]. In the past decades, several experiences showed that NE is associated with clinical outcomes in different bronchiectasis cohorts [66, 74, 75]. Tsang and coworkers showed a correlation between sputum elastase, radiological involvement, and functional markers in a sample of 30 bronchiectasis patients [75]. However, the heterogeneity of measurement assays for NE among different cohorts and the lack of relevant follow-up periods may have affected the findings. Recently, NE underwent a prospective validation in a large cohort of patients followed over 3 years. This sample was divided into three groups according to negative, intermediate, and high elastase activity levels. NE activity was independently associated with lung function decline over 3 years with a loss of 56.4 ml per year in the high activity group (+20.8 ml in comparison with negative-NE group). In addition, NE was also independently associated with the risk of future exacerbations, shorter time to next exacerbation, and all-cause mortality [17].

In a recent evaluation of clinical phenotypes, the phenotype of patients with chronic infection with *P. aeruginosa* or other bacteria was associated with both higher NE concentrations and worse clinical outcome [70]. This finding is consistent with a meta-analysis by Finch which showed that *P. aeruginosa* chronic infection is associated with a threefold increased risk of death and an increase in hospital admissions and exacerbations [76].

Future clinical trials are needed to validate cut-off of NE activity and implement NE as a useful biomarker in the clinical management of bronchiectasis patients.

Local or systemic antibiotic therapy and consequent reduction of bacterial load has been hypothesized to decrease sputum NE levels. Previous findings suggest that short oral courses of antibiotic therapy are effective in reducing NE activity in sputum [6, 67, 71]. Chalmers and Murray proved that long-term nebulized gentamycin significantly reduces free elastase activity in sputum, as well as sputum MPO levels and bacterial load [67, 77].

Roxithromycin treatment may reduce sputum NE in bronchiectasis patients as recently demonstrated by Liu in an open-label 6-month study. These findings demonstrate that macrolide therapy may be beneficial in bronchiectasis and may contribute to the understanding of underlying mechanisms [14, 78].

Selected anti-inflammatory drugs have also been tested as indirect modulators of neutrophilic inflammation in bronchiectasis. In a study by Llewellyn-Jones, a 4-week treatment with indomethacin appeared to have no effect on lung inflammation, as assessed by no significant difference in neutrophil and NE activity within the sputum [15].

### NE as a target of treatment

**Fig. 2:**
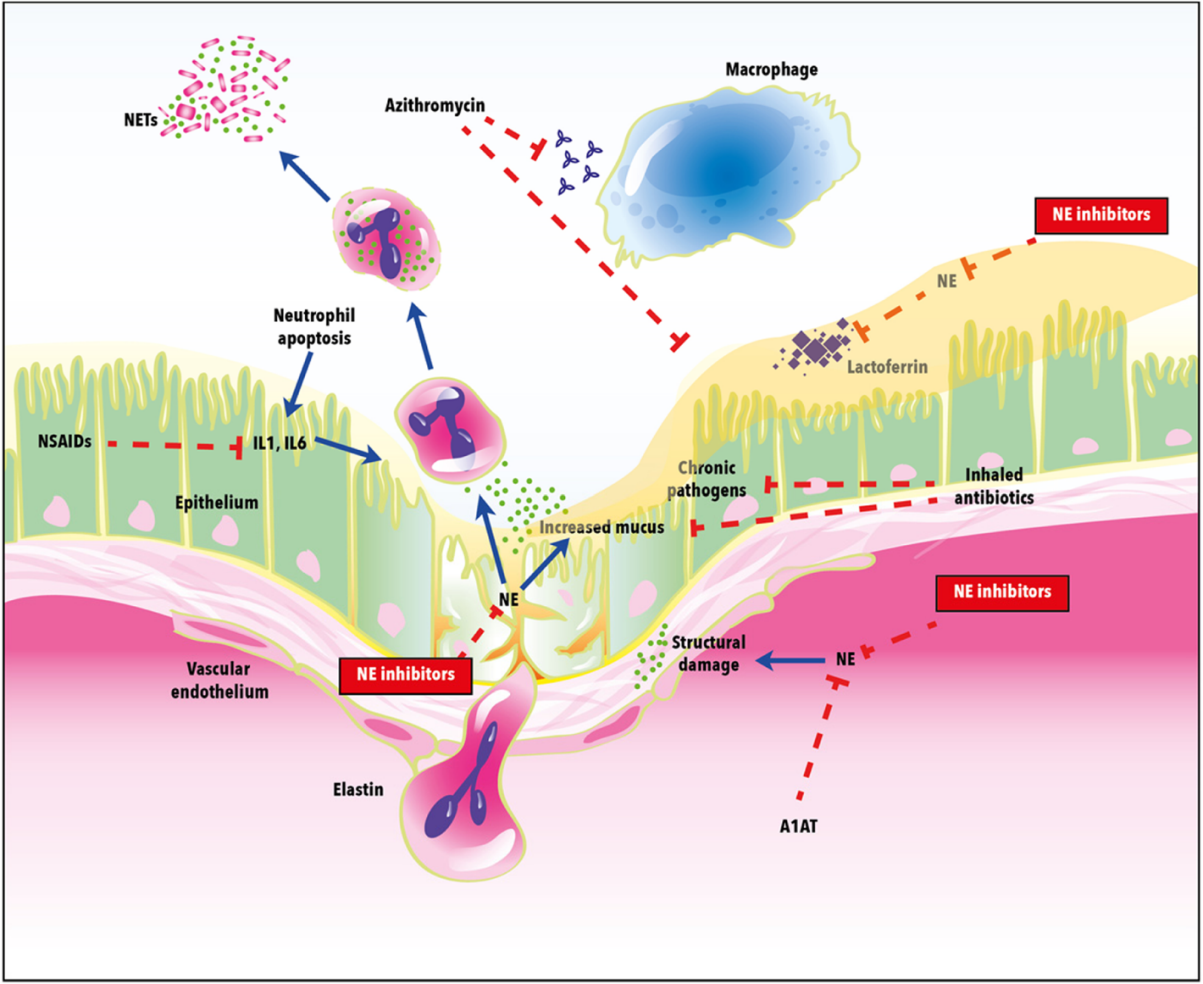
Neutrophil elastase: mechanisms of action and treatment targets in bronchiectasis. Definitions: NE = neutrophil elastase; A1AT = alpha-1 anti-trypsin; NSAIDS = non-steroidal anti-inflammatory drugs

Since NE is a major driver of neutrophilic inflammation in bronchiectasis, it has been proposed to tailor treatment aimed to its direct or indirect inhibition, see Fig. 2.

AZD9668 is a NE inhibitor previously investigated in chronic respiratory diseases, including COPD and CF, with inconclusive results [34, 44, 45]. In a small phase II double-blind trial conducted in 10 different centres, 38 patients were randomized to 4 weeks of oral AZD9668 twice daily. AZD9668 increased FEV_1_ (by about 100 mL) and slowed vital capacity (by about 130 mL) compared with the placebo group. A trend in reduction of most inflammatory biomarkers was also reported (IL-6 and IL-8). There were no differences between AZD9668 and placebo arms regarding sputum quantity, symptoms, and quality of life. The Authors suggested larger studies of longer duration to assess beneficial effects [55, 79].

BAY 85–8501 is a selective NE inhibitor that has shown efficacy in pre-clinical pharmacological and animal models [80]. A phase II randomized parallel group study assessed the safety and tolerability of 28-day oral administration of BAY 85–8501 in 94 bronchiectasis patients. BAY 85–8501 was generally well tolerated and there were no significant changes in lung function, 24 h sputum quantity, NE activity and concentration, and other biomarkers including urine desmosine [81]. Short duration of treatment and inadequate drug concentration at the target site may be the cause of this lack of efficacy. Regarding this last point, it is reasonable that the inhaled approach instead of the oral administration could allow reaching higher lung concentrations with limited systemic exposure and consequent side effects, thus permitting to counteract that elevated amount of active NE found in the patient’s airways.

With this rationale, CHF6333 is the first inhaled NE inhibitor under development for the treatment of NE-driven lung diseases as dry powder inhaler. In in vitro assays, CHF6333 is highly potent in inhibiting human NE (IC_50_ = 0.2 nM) with good selectivity against other proteases. In addition, CHF6333 significantly reduces lung neutrophil recruitment induced by cigarette smoke exposure in mice and reduces both lung tissue infection and inflammation when administered intratracheally to *P. aeruginosa* lung infected rats for 7 days [82].

A phase I trial to investigate safety and pharmacokinetics on healthy male subjects is still in phase of recruitment (clinicaltrial.gov ID NCT03056326).

## Conclusions

Sputum NE has proved useful as an inflammatory marker both in stable state bronchiectasis and during exacerbations and local or systemic antibiotic treatment. NE has also been associated with risk of exacerbation, time to next exacerbation and all-cause mortality. Inhibition of NE as a specific target of treatment in bronchiectasis is at a very early stage. Future interventional studies should evaluate safety and efficacy for new molecules and formulations.

## References

[1] Mantovani A, Cassatella MA, Costantini C, Jaillon S (2011). Neutrophils in the activation and regulation of innate and adaptive immunity. Nat Rev Immunol.

[2] Silvestre-Roig C, Hidalgo A, Soehnlein O (2016). Neutrophil heterogeneity: implications for homeostasis and pathogenesis. Blood.

[3] Weiss SJ. Tissue destruction by neutrophils. N Engl J Med. 1989 Feb9;320(6):365–376. 10.1056/NEJM198902093200606 2536474

[4] Weissmann G, Smolen JE, Korchak HM (1980). Release of inflammatory mediators from stimulated neutrophils. N Engl J Med.

[5] Tosi MF, Zakem H, Berger M (1990). Neutrophil elastase cleaves C3bi on opsonised pseudomonas as well as CR1 on neutrophils to create a functionally important opsonin receptor mismatch. J Clin Invest.

[6] Smallman LA, Hill SL, Stockley RA (1984). Reduction of ciliary beat frequency in vitro by sputum from patients with bronchiectasis: a serine proteinase effect. Thorax.

[7] Amitani R, Wilson R, Rutman A, Read R, Ward C, Burnett D, Stockley RA, Cole PJ (1991). Effects of human neutrophil elastase and Pseudomonas Aeruginosa proteinases on human respiratory epithelium. Am J Respir Cell Mol Biol.

[8] Cech P, Lehrer RI (1984). Phagolysosomal pH of human neutrophils. Blood.

[9] Owen CA, Campbell MA, Sannes PL, Boukedes SS, Campbell EJ (1995). Cell surface-bound elastase and cathepsin G on human neutrophils: a novel, non-oxidative mechanism by which neutrophils focus and preserve catalytic activity of serine proteinases. J Cell Biol.

[10] Brinkmann V, Reichard U, Goosmann C, Fauler B, Uhlemann Y, Weiss DS, Weinrauch Y, Zychlinsky A (2004). Neutrophil extracellular traps kill bacteria. Science.

[11] Sandhaus RA, Turino G (2013). Neutrophil elastase-mediated lung disease. COPD..

[12] Chan SC, Shum DK, Ip MS. Sputum sol neutrophil elastase activity in bronchiectasis: differential modulation by syndecan-1. Am J Respir Crit Care Med 2003 Jul 15;168(2):192-8. Epub 2003 Apr 17. 10.1164/rccm.200208-829OC 12702549

[13] Korkmaz B, Moreau T, Gauthier F. Neutrophil elastase, proteinase 3 and cathepsin G: physicochemical properties, activity and physiopathological functions. Biochimie 2008 Feb;90(2):227-42. Epub 2007 Oct 25. 10.1016/j.biochi.2007.10.009 18021746

[14] Liu J, Zhong X, He Z, Wei L, Zheng X, Zhang J, Bai J, Zhong W, Zhong D (2014). Effect of low-dose, long-term roxithromycin on airway inflammation and remodeling of stable noncystic fibrosis bronchiectasis. Mediat Inflamm.

[15] Llewellyn-Jones CG, Johnson MM, Mitchell JL, Pye A, Okafor VC, Hill SL, Stockley RA (1995). In vivo study of indomethacin in bronchiectasis: effect on neutrophil function and lung secretion. Eur Respir J.

[16] Hill AT, Campbell EJ, Hill SL, Bayley DL, Stockley RA (2000). Association between airway bacterial load and markers of airway inflammation in patients with stable chronic bronchitis. Am J Med.

[17] Chalmers JD, Moffitt KL, Suarez-Cuartin G, Sibila O, Finch S, Furrie E, Dicker A, Wrobel K, Elborn JS, Walker B, Martin SL, Marshall SE, Huang JT, Fardon TC (2017). Neutrophil Elastase activity is associated with exacerbations and lung function decline in Bronchiectasis. Am J Respir Crit Care Med.

[18] Stockley RA, Hill SL, Morrison HM, Starkie CM (1984). Elastolytic activity of sputum and its relation to purulence and to lung function in patients with bronchiectasis. Thorax.

[19] Starcher B, Scott M (1992). Fractionation of urine to allow desmosine analysis by radioimmunoassay. Ann Clin Biochem.

[20] Albarbarawi O, Barton A, Miller D, McSharry C, Chaudhuri R, Thomson NC, Palmer CN, Devereux G, Huang JT (2013). Characterization and validation of an isotope-dilution LC-MS/MS method for quantification of total desmosine and isodesmosine in plasma and serum. Bioanalysis.

[21] Viglio S, Valentini G, Chiarelli L, Zanaboni G, Cetta G, Iadarola P (1999). Micellar electrokinetic chromatography as a complementary method to sodium dodecyl sulfate-polyacrylamide gel electrophoresis for studying limited proteolysis of proteins. Electrophoresis.

[22] Khan TZ, Wagener JS, Bost T, Martinez J, Accurso FJ, Riches DW (1995). Early pulmonary inflammation in infants with cystic fibrosis. Am J Respir Crit Care Med.

[23] Sly PD, Brennan S, Gangell C, de Klerk N, Murray C, Mott L, Stick SM, Robinson PJ, Robertson CF, Ranganathan SC (2009). Australian respiratory early surveillance team for cystic fibrosis (AREST-CF). Lung disease at diagnosis in infants with cystic fibrosis detected by newborn screening. Am J Respir Crit Care Med.

[24] Sagel SD, Wagner BD, Anthony MM, Emmett P, Zemanick ET (2012). Sputum biomarkers of inflammation and lung function decline in children with cystic fibrosis. Am J Respir Crit Care Med.

[25] Park JA, He F, Martin LD, Li Y, Chorley BN, Adler KB (2005). Human neutrophil elastase induces hypersecretion of mucin from well-differentiated human bronchial epithelial cells in vitro via a protein kinase C{delta}-mediated mechanism. Am J Pathol.

[26] Rubio F, Cooley J, Accurso FJ, Remold-O'Donnell E (2004). Linkage of neutrophil serine proteases and decreased surfactant protein-a (SP-A) levels in inflammatory lung disease. Thorax.

[27] Van den Steen PE, Proost P, Wuyts A, Van Damme J, Opdenakker G (2000). Neutrophil gelatinase B potentiates interleukin-8 tenfold by aminoterminal processing,whereas it degrades CTAP-III, PF-4, and GRO-alpha and leaves RANTES and MCP-2 intact. Blood.

[28] Rogan MP, Taggart CC, Greene CM, Murphy PG, O'Neill SJ, McElvaney NG (2004). Loss of microbicidal activity and increased formation of biofilm due to decreased lactoferrin activity in patients with cystic fibrosis. J Infect Dis.

[29] Guyot N, Butler MW, McNally P, Weldon S, Greene CM, Levine RL, O’Neill SJ, Taggart CC, McElvaney NG (2008). Elafin, an elastase-specific inhibitor, is cleaved by its cognate enzyme neutrophil elastase in sputum from individuals with cystic fibrosis. J Biol Chem.

[30] Döring G, Frank F, Boudier C, Herbert S, Fleischer B, Bellon G (1995). Cleavage of lymphocyte surface antigens CD2, CD4, and CD8 by polymorphonuclear leukocyte elastase and cathepsin G in patients with cystic fibrosis. J Immunol.

[31] Hartl D, Latzin P, Hordijk P, Marcos V, Rudolph C, Woischnik M, Krauss-Etschmann S, Koller B, Reinhardt D, Roscher AA, Roos D, Griese M (2007). Cleavage of CXCR1 on neutrophils disables bacterial killing in cystic fibrosis in lung disease. Nat Med.

[32] Gentzsch M, Dang H, Dang Y, Garcia-Caballero A, Suchindran H, Boucher RC, Stutts MJ (2010). The cystic fibrosis transmembrane conductance regulator impende proteolytic stimulation of the epithelial Na+ channel. J Biol Chem.

[33] Le Gars M, Descamps D, Roussel D, Saussereau E, Guillot L, Ruffin M, Tabary O, Hong SS, Boulanger P, Paulais M, Malleret L, Belaaouaj A, Edelman A, Huerre M, Chignard M, Sallenave JM (2013). Neutrophil elastase degrades cystic fibrosis transmembrane conductance regulator via calpains and disables channel function in vitro and in vivo. Am J Respir Crit Care Med.

[34] Elborn JS, Perrett J, Forsman-Semb K, Marks-Konczalik J, Gunawardena K, Entwistle N (2012). Efficacy, safety and effect on biomarkers of AZD9668 in cystic fibrosis. Eur Respir J.

[35] Griese M, Latzin P, Kappler M, Weckerle K, Heinzlmaier T, Bernhardt T, Hartl D (2007). alpha1-antitrypsin inhalation reduces airway inflammation in cystic fibrosis patients. Eur Respir J.

[36] Stănescu D, Sanna A, Veriter C, Kostianev S, Calcagni PG, Fabbri LM, Maestrelli P (1996). Airways obstruction, chronic expectoration, and rapid decline of FEV1 in smokers are associated with increased levels of sputum neutrophils. Thorax.

[37] Barnes PJ (2009). The cytokine network in chronic obstructive pulmonary disease. Am J Respir Cell Mol Biol.

[38] Richens TR, Linderman DJ, Horstmann SA, Lambert C, Xiao YQ, Keith RL, Boé DM, Morimoto K, Bowler RP, Day BJ, Janssen WJ, Henson PM, Vandivier RW (2009). Cigarette smoke impairs clearance of apoptotic cells through oxidant-dependent activation of RhoA. Am J Respir Crit Care Med.

[39] Damiano VV, Tsang A, Kucich U, Abrams WR, Rosenbloom J, Kimbel P, Fallahnejad M, Weinbaum G (1986). Immunolocalization of elastase in human emphysematous lungs. J Clin Invest.

[40] Lungarella G, Cavarra E, Lucattelli M, Martorana PA (2008). The dual role of neutrophil elastase in lung destruction and repair. Int J Biochem Cell Biol.

[41] Shapiro SD, Goldstein NM, Houghton AM, Kobayashi DK, Kelley D, Belaaouaj A (2003). Neutrophil elastase contributes to cigarette smoke-induced emphysema in mice. Am J Pathol.

[42] Paone G, Conti V, Vestri A, Leone A, Puglisi G, Benassi F, Brunetti G, Schmid G, Cammarella I, Terzano C (2011). Analysis of sputum markers in the evaluation of lung inflammation and functional impairment in symptomatic smokers and COPD patients. Dis Markers.

[43] Stevens T, Ekholm K, Gränse M, Lindahl M, Kozma V, Jungar C, Ottosson T, Falk-Håkansson H, Churg A, Wright JL, Lal H, Sanfridson A (2011). AZD9668: pharmacological characterization of a novel oral inhibitor of neutrophil elastase. J Pharmacol Exp Ther.

[44] Kuna P, Jenkins M, O'Brien CD, Fahy WA (2012). AZD9668, a neutrophil elastase inhibitor, plus ongoing budesonide/formoterol in patients with COPD. Respir Med.

[45] Vogelmeier C, Aquino TO, O'Brien CD, Perrett J, Gunawardena KA (2012). A randomised, placebo-controlled, dose-finding study of AZD9668, an oral inhibitor of neutrophil elastase, in patients with chronic obstructive pulmonary disease treated with tiotropium. COPD.

[46] Chapman KR, Burdon JG, Piitulainen E, Sandhaus RA, Seersholm N, Stocks JM, Stoel BC, Huang L, Yao Z, Edelman JM, NG ME (2015). RAPID trial study group. Intravenous augmentation treatment and lung density in severe α1 antitrypsin deficiency (RAPID): a randomised, double-blind, placebo-controlled trial. Lancet.

[47] Gibson PG, Simpson JL, Saltos N (2001). Heterogeneity of airway inflammation in persistent asthma: evidence of neutrophilic inflammation and increased sputum interleukin-8. Chest.

[48] Louis R, Djukanovic R (2006). Is the neutrophil a worthy target in severe asthma and chronic obstructive pulmonary disease?. Clin Exp Allergy.

[49] Simpson JL, Scott RJ, Boyle MJ, Gibson PG (2005). Differential proteolytic enzyme activity in eosinophilic and neutrophilic asthma. Am J Respir Crit Care Med.

[50] Chalmers JD, Aliberti S, Blasi F (2015). Management of bronchiectasis in adults. Eur Respir J.

[51] Aliberti S, Masefield S, Polverino E, De Soyza A, Loebinger MR, Menendez R, Ringshausen FC, Vendrell M, Powell P, Chalmers JD, EMBARC Study Group (2016). Research priorities in bronchiectasis: a consensus statement from the EMBARC clinical research collaboration. Eur Respir J.

[52] Amati F, Franceschi E, Gramegna A, Chalmers JD, Aliberti S (2017). Investigating the etiology of Bronchiectasis: you do not find what you do not look for. Respiration.

[53] Dente FL, Bilotta M, Bartoli ML (2015). Neutrophilic bronchial inflammation correlates with clinical and functional findings in patients with noncystic fibrosis Bronchiectasis. Mediat Inflamm.

[54] Moher D, Liberati A, Tetzlaff J, Altman DG; PRISMA group. Preferred reporting items for systematic reviews and meta-analyses: the PRISMA statement. Ann Intern Med 2009 Aug 18;151(4):264-9, W64. 10.7326/0003-4819-151-4-200908180-00135 19622511

[55] Stockley R, De Soyza A, Gunawardena K, Perrett J, Forsman-Semb K, Entwistle N, Snell N (2013). Phase II study of a neutrophil elastase inhibitor (AZD9668) in patients with bronchiectasis. Respir Med.

[56] Nakamura H, Abe S, Shibata Y, Yuki H, Suzuki H, Saito H, Sata M, Kato S, Tomoike H (1997). Elevated levels of cytokeratin 19 in the bronchoalveolar lavage fluid of patients with chronic airway inflammatory diseases--a specific marker for bronchial epithelial injury. Am J Respir Crit Care Med.

[57] Gaga M, Bentley AM, Humbert M, Barkans J, O'Brien F, Wathen CG, Kay AB, Durham SR (1998). Increases in CD4+ T lymphocytes, macrophages, neutrophils and interleukin 8 positive cells in the airways of patients with bronchiectasis. Thorax.

[58] Vandivier RW, Fadok VA, Hoffmann PR, Bratton DL, Penvari C, Brown KK, Brain JD, Accurso FJ, Henson PM (2002). Elastase-mediated phosphatidylserine receptor cleavage impairs apoptotic cell clearance in cystic fibrosis and bronchiectasis. J Clin Invest.

[59] Zheng L, Shum H, Tipoe GL, Leung R, Lam WK, Ooi GC, Tsang KW (2001). Macrophages, neutrophils and tumour necrosis factor-alpha expression in bronchiectatic airways in vivo. Respir Med.

[60] Fujita J, Nakamura H, Yamagishi Y, Yamaji Y, Shiotani T, Irino S (1992). Elevation of plasma truncated elastase alpha 1-proteinase inhibitor complexes in patients with inflammatory lung diseases. Chest.

[61] Chan SC, Leung VO, Ip MS, Shum DK (2009). Shed syndecan-1 restricts neutrophil elastase from alpha1-antitrypsin in neutrophilic airway inflammation. Am J Respir Cell Mol Biol.

[62] Angrill J, Agustí C, De Celis R, Filella X, Rañó A, Elena M, De La Bellacasa JP, Xaubet A, Torres A (2001). Bronchial inflammation and colonization in patients with clinically stable bronchiectasis. Am J Respir Crit Care Med.

[63] Lloberes P, Montserrat E, Montserrat JM, Picado C (1992). Sputum sol phase proteins and elastase activity in patients with clinically stable bronchiectasis. Thorax.

[64] Ichikawa W, Ogushi F, Tani K, Maniwa K, Kamada M, Ohmoto Y, Sakatani M, Sone S (1999). Characterization of immunoglobulin binding factor in sputum from patients with chronic airway diseases. Respirology.

[65] Stockley RA, Bayley D, Hill SL, Hill AT, Crooks S, Campbell EJ (2001). Assessment of airway neutrophils by sputum colour: correlation with airways inflammation. Thorax.

[66] Goeminne PC, Vandooren J, Moelants EA, Decraene A, Rabaey E, Pauwels A, Seys S, Opdenakker G, Proost P, Dupont LJ (2014). The sputum colour chart as a predictor of lung inflammation, proteolysis and damage in non-cystic fibrosis bronchiectasis: a case-control analysis. Respirology.

[67] Chalmers JD, Smith MP, McHugh BJ, Doherty C, Govan JR, Hill AT (2012). Short- and long term antibiotic treatment reduces airway and systemic inflammation in non-cystic fibrosis bronchiectasis. Am J Respir Crit Care Med.

[68] Schaaf B, Wieghorst A, Aries SP, Dalhoff K, Braun J (2000). Neutrophil inflammation and activation in bronchiectasis: comparison with pneumonia and idiopathic pulmonary fibrosis. Respiration.

[69] Zheng L, Tipoe G, Lam WK, Ho JC, Shum I, Ooi GC, Leung R, Tsang KW (2000). Endothelin-1 in stable bronchiectasis. Eur Respir J.

[70] Aliberti S, Lonni S, Dore S, McDonnell MJ, Goeminne PC, Dimakou K, Fardon TC, Rutherford R, Pesci A, Restrepo MI, Sotgiu G, Chalmers JD (2016). Clinical phenotypes in adult patients with bronchiectasis. Eur Respir J.

[71] Ip M, Shum D, Lauder I, Lam WK, So SY (1993). Effect of antibiotics on sputum inflammatory contents in acute exacerbations of bronchiectasis. Respir Med.

[72] Watt AP, Brown V, Courtney J, Kelly M, Garske L, Elborn JS, Ennis M (2004). Neutrophil apoptosis, proinflammatory mediators and cell counts in bronchiectasis. Thorax.

[73] Mandal P, Morice AH, Chalmers JD, Hill AT (2013). Symptoms of airway reflux predict exacerbations and quality of life in bronchiectasis. Respir Med.

[74] Sepper R, Konttinen YT, Ingman T, Sorsa T (1995). Presence, activities, and molecular forms of cathepsin G, elastase, alpha 1-antitrypsin, and alpha 1-antichymotrypsin in bronchiectasis. J Clin Immunol.

[75] Tsang KW, Chan K, Ho P, Zheng L, Ooi GC, Ho JC, Lam W (2000). Sputum elastase in steady-state bronchiectasis. Chest.

[76] Finch S, McDonnell MJ, Abo-Leyah H, Aliberti S, Chalmers JD (2015). A comprehensive analysis of the impact of Pseudomonas Aeruginosa colonization on prognosis in adult Bronchiectasis. Ann Am Thorac Soc.

[77] Murray MP, Govan JR, Doherty CJ, Simpson AJ, Wilkinson TS, Chalmers JD, Greening AP, Haslett C, Hill AT (2011). A randomized controlled trial of nebulized gentamicin in non-cystic fibrosis bronchiectasis. Am J Respir Crit Care Med.

[78] Nakamura H, Fujishima S, Inoue T, Ohkubo Y, Soejima K, Waki Y, Mori M, Urano T, Sakamaki F, Tasaka S, Ishizaka A, Kanazawa M, Yamaguchi K (1999). Clinical and immunoregulatory effects of roxithromycin therapy for chronic respiratory tract infection. Eur Respir J.

[79] Gunawardena KA, Gullstrand H, Perrett J (2013). Pharmacokinetics and safety of AZD9668, an oral neutrophil elastase inhibitor, in healthy volunteers and patients with COPD. Int J Clin Pharmacol Ther.

[80] von Nussbaum F, Li VM, Allerheiligen S, Anlauf S, Bärfacker L, Bechem M, Delbeck M, Fitzgerald MF, Gerisch M, Gielen-Haertwig H, Haning H, Karthaus D, Lang D, Lustig K, Meibom D, Mittendorf J, Rosentreter U, Schäfer M, Schäfer S, Schamberger J, Telan LA, Tersteegen A (2015). Freezing the bioactive conformation to boost potency: the identification of BAY 85-8501, a selective and potent inhibitor of human Neutrophil Elastase for pulmonary diseases. ChemMedChem.

[81] Watz H, Pedersen F, Kirsten A, Nagelschmitz J, Bandel TJ, Schwers S, Rabe K. Safety and tolerability of the NE inhibitor BAY 85-8501 in patients with non-CF bronchiectasis. ERS International Congress 2016.

[82] Miglietta D, Carnini C, Puviani V, Finch H, Fox C, Fitzgerald M, Patacchini R, Civelli M, Villetti G. Pharmacological characterization of CHF6333, a novel potent inhaled inhibitor of neutrophil elastase. ERS International Congress 2016.

